# Changes in α-Farnesene and Phenolic Metabolism and the Expression of Associated Genes during the Development of Superficial Scald in Two Distinct Pear Cultivars

**DOI:** 10.3390/ijms232012088

**Published:** 2022-10-11

**Authors:** Jingang He, Yunxiao Feng, Yudou Cheng, Thirupathi Karuppanapandian, Jinxiao Wang, Junfeng Guan

**Affiliations:** 1 Institute of Biotechnology and Food Science, Hebei Academy of Agriculture and Forestry Sciences, Shijiazhuang 050051, China; 2 Hebei Plant Genetic Engineering Center, Shijiazhuang 050051, China; 3 Department of Horticultural Science, Stellenbosch University, Stellenbosch 7602, South Africa

**Keywords:** pear, α-farnesene, conjugated trienols, phenolics, superficial scald

## Abstract

Superficial scald is a postharvest physiological disorder that occurs in pear during and after cold storage. In this study, the superficial scald index; α-farnesene and its oxidation products, conjugated trienols (CTols); phenolic content; and the expression of its related genes were investigated in two different pear cultivars, ‘Wujiuxiang’ (*Pyrus communis* L.) and ‘Yali’ (*Pyrus bretschneideri* R.), following 115 days of cold storage at 0 °C followed by 7 days of shelf life at 20 °C. The results indicated that the superficial scald occurred after 115 days of cold storage and became more severe during the shelf life of the ‘Wujiuxiang’ pear, whereas no scald was observed in ‘Yali’. The α-farnesene levels increased rapidly at first and then decreased, while the CTols contents increased significantly in ‘Wujiuxiang’ as compared to ‘Yali’, and the expression levels of the genes involved in α-farnesene and CTols metabolism (*HMGR1*, *HMGR2*, *GSTU7*, *GPX5*, and *GPX6)*, as well as the phenolic synthesis (*PAL1*, *PAL2*, *C4H1*, *4CL2*, *C3H*, and *ANR*) of the peel, were significantly up-regulated at the onset of the superficial scald. In addition, the relative conductivity and contents of catechin and epicatechin were higher, and the expression level of the laccase gene (*LAC7*) significantly increased with the development of superficial scald, while lower contents of chlorogenic acid, arbutin, and isorhamnetin-3-3-glucoside, as well as the lower expression levels of a phenolic-synthesis-related gene (*C4H3*) and polyphenol oxidase genes (*PPO1* and *PPO5*), were noticed in ‘Wujiuxiang’ as compared to ‘Yali’. The results indicated that the onset and progression of superficial scald were associated with the accumulation of CTols, cell membrane breakdown, and higher catechin, epicatechin, and rutin contents, as well as the expression of associated genes of the peels of pear fruit.

## 1. Introduction

Superficial scald is a physiological disorder that affects pear fruit quality during and after long-term cold storage, resulting a great loss of the fruit’s commercial value. The α-farnesene and its oxidation products, conjugated trienols (CTols), are closely associated with the development of superficial scald [1,2,3,4,5,6,7,8,9,10,11]. Although the metabolic pathways involved in α-farnesene synthesis and its oxidation process have yet to be fully elucidated [12], it is known that the higher gene expression of hydroxymethylglutaryl-CoA reductase (*HMGR*), farnesyl pyrophosphate synthase (*FPP*), α-farnesene synthase (*AFS*), glutathione S-transferases (*GST*), and glutathione peroxidase (*GPX*) are positively correlated with the development of superficial scald [1,6,9,13,14,15,16,17,18,19]. Furthermore, the cell membrane damage caused by the accumulation of reactive oxygen species (ROS), phenolic compounds (especially chlorogenic acid and epicatechin), and their oxidation process, catalyzed by polyphenol oxidase (PPO) and laccase (LAC), are linked to the development of superficial scald [6,11,12,15,20,21,22]. It has been showed that CTols are more essential in the development of superficial scald than α-farnesene [4], and the ROS metabolism characteristics vary depending on the cultivar [23]. However, there are few studies comparing cultivars with varying degrees of susceptibility to superficial scald [4,14,19,22,23,24].

In this work, two pear cultivars displaying distinct levels of superficial scald susceptibility were investigated. The ‘Yali’ pear (*Pyrus bretschneideri* R.), a famous cultivar in China, develops little to no signs of superficial scald during 4 months of cold storage [21]. In contrast, the ‘Wujiuxiang’ pear (*Pyrus communis* L.) is a hybrid progeny of ‘Yali’ and ‘Bartlett’ (*Pyrus communis* L.), and it is prone to developing marked superficial scald during approximately 4 months of cold storage [6,25,26,27]. The objective of this study is to elucidate the mechanism of superficial scald in pears with different genetic backgrounds and levels of sensitivity to superficial scald and, furthermore, to explore the expression patterns of genes associated with α-farnesene, CTols, and the phenolic metabolism pathway, as well as the cell membrane permeability of the peels.

## 2. Results

### 2.1. Changes in the Superficial Scald Index

**Figure 1:**
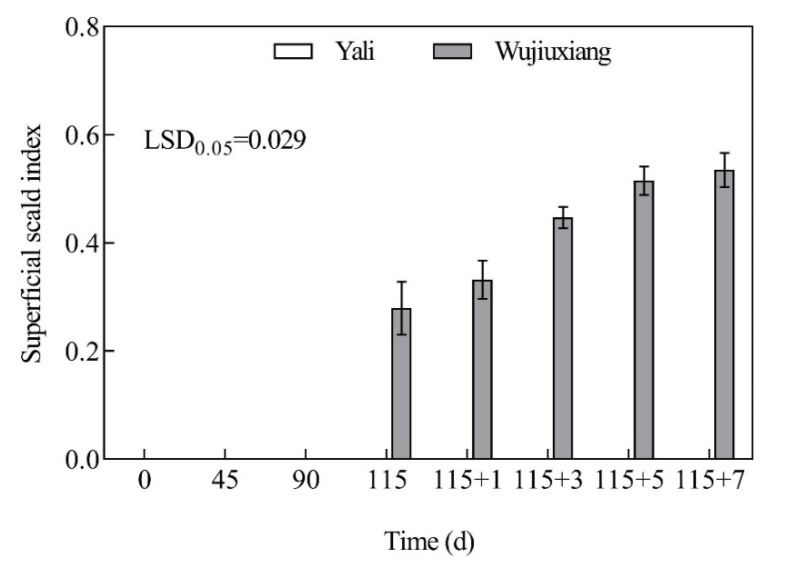
Superficial scald indexes of ‘Yali’ and ‘Wujiuxiang’ pear during cold storage and the shelf life period. Vertical bars represent standard errors and the LSD values show significant differences at the 0.05 level. Note: 115 + 1, 115 + 3, 115 + 5, and 115 + 7 represent 1, 3, 5, and 7 days of shelf life at room temperature (20 °C) after cold storage at 0 °C for 115 days.

In ‘Wujiuxiang’, superficial scald was noticed after 115 daysof cold storage, and the severity of the scald markedly increased after the shelf life period, with the superficial scald index increased by 82.48% at day 115 + 7 compared to day 115 (Figure 1). However, during cold storage and the shelf life period, there was no scald found in ‘Yali’ (Figure 1).

### 2.2. Changes in the Levels of α-Farnesene and CTols and Their Related Gene Expression

**Figure 2:**
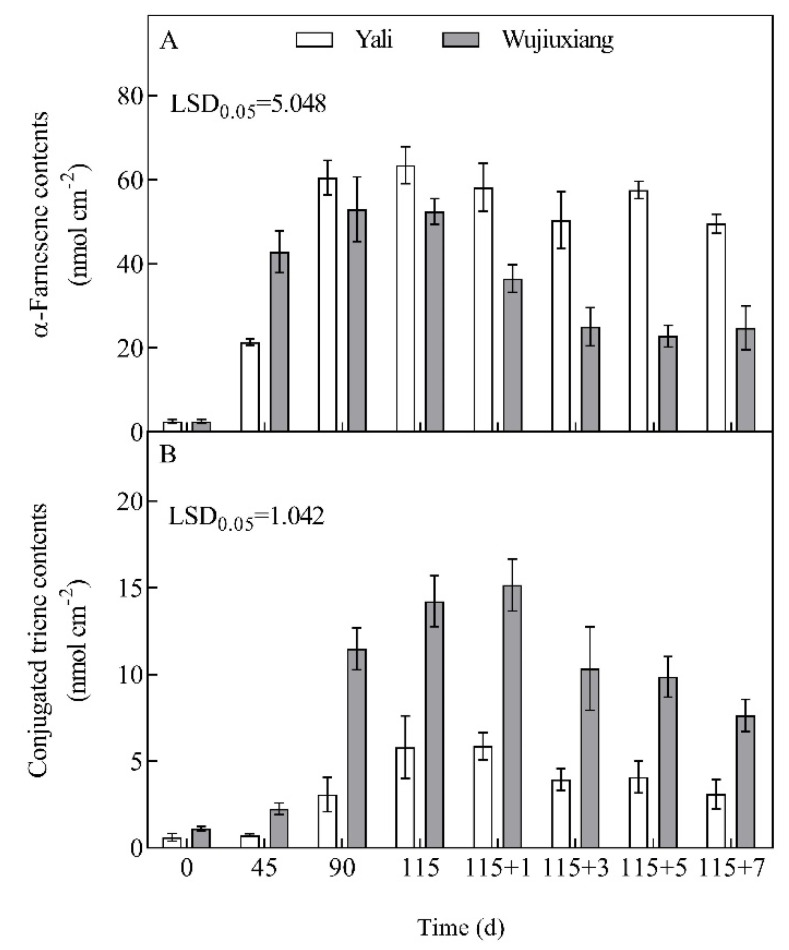
Contents of α-farnesene (**A**) and CTols (**B**) in the peels of ‘Yali’ and ‘Wujiuxiang’ pear during cold storage and the shelf life period. Vertical bars represent standard errors and LSD values represent significant differences at the level of 0.05. Note: 115 + 1, 115 + 3, 115 + 5, and 115 + 7 indicate 1, 3, 5 and 7 days of shelf life at room temperature (20 °C) after cold storage at 0 °C for 115 days.

In ‘Wujiuxiang’, α-farnesene had accumulated significantly after 90 days of cold storage and subsequently began to fall in the shelf life period. However, the α-farnesene levels were higher in ‘Yali’ than in ‘Wujiuxiang’ after 90 days of cold storage. With the extension of the cold storage duration, the peel gradually accumulated CTols, which peaked on the first day (115 + 1) of the shelf life and subsequently declined in ‘Wujiuxiang’, which was significantly higher in terms of the CTols level than ‘Yali’ (Figure 2).

**Figure 3:**
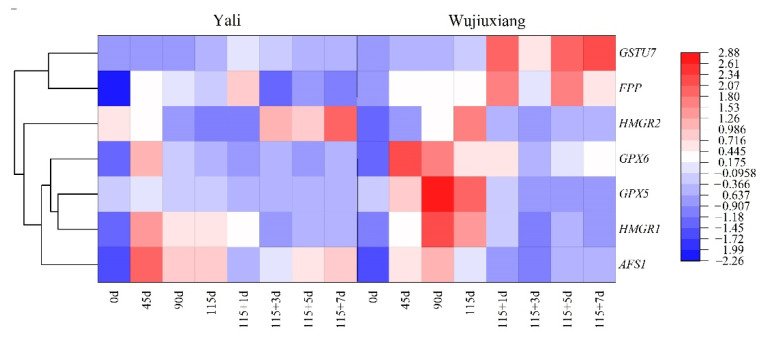
Expression profiles of α-farnesene and CTols biosynthesis-related genes of the ‘Yali’ and ‘Wujiuxiang’ pears during cold storage and the shelf life period. Note: 115 + 1, 115 + 3, 115 + 5, and 115 + 7 indicate 1, 3, 5 and 7 days of shelf life at room temperature (20 °C) after cold storage at 0 °C for 115 days. Red and high positive values indicate high gene expression, while blue and low negative values indicate low gene expression.

The expression of *AFS1* was sharply upregulated during the early stage of cold storage, and then dropped and elevated slightly during the shelf life of the two cultivars (Figure 3), but the expression level of *AFS1* in ‘Yali’ was higher than in ‘Wujiuxiang’, except for the observation at 90 d. The expression patterns of *HMGR1* and *HMGR2* were slightly different, with ‘Wujiuxiang’ having considerably greater expression levels than ‘Yali’ after 90 days and 115 days of cold storage, and afterwards, the difference in the *HMGR1* expression between the two cultivars was diminished. The *HMGR2* expression of ‘Wujiuxiang’ was significantly lower than that of ‘Yali’ at days 115 + 3, 115 + 5, and 115 + 7. The expression of *FPP* in ‘Wujiuxiang’ was higher at the beginning of the cold storage period, but there was no significant difference between the two cultivars during the later period of cold storage. Nevertheless, it was higher in ‘Wujiuxiang’ than in ‘Yali’ at days 115 + 3, 115 + 5, and 115 + 7 (Figure 3).

The expression of *GSTU7* increased gradually during cold storage, and then quickly decreased at the beginning of the shelf life period (115 + 1 d). Relatively speaking, the *GSTU7* expression level of ‘Yali’ was significantly lower than that of ‘Wujiuxiang’. The expression level of *GPX5* in ‘Yali’ decreased gradually, but it peaked after 90 days of cold storage, and the expression level was significantly higher during cold storage in ‘Wujiuxiang’ than in ‘Yali’, except at day 3 of the shelf life (115 + 3). With the exception of day 3 of the shelf life (115 + 3), the expression level of *GPX6* in the two pear varieties increased distinctly in the early stages of cold storage and thereafter decreased, and it was substantially higher in ‘Wujiuxiang’ than in ‘Yali’ (Figure 3). The specific expression values are shown in Supplementary Table S2.

### 2.3. Changes in the Phenolic Content and Their Related Gene Expression

**Figure 4:**
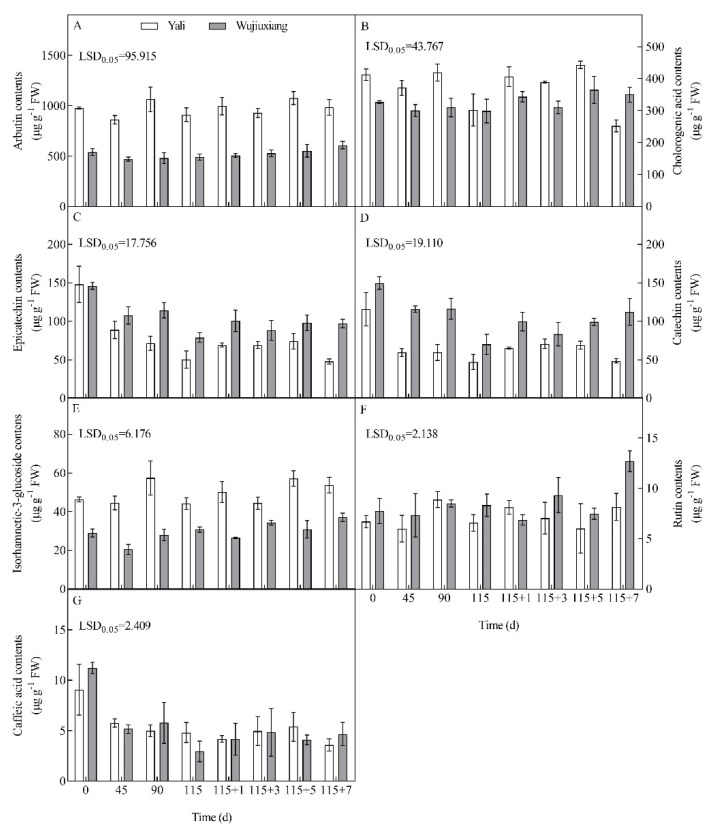
Contents of arbutin (**A**), chlorogenic acid (**B**), epicatechin (**C**), catechin (**D**), isorhamnetic-3-glucoside (**E**), rutin (**F**), and caffeic acid (**G**) in the peels of ‘Yali’ and ‘Wujiuxiang’ pears during cold storage and the shelf life. The vertical bar represents the standard error while the LSD values show significant differences at the 0.05 level. Note: 115 + 1, 115 + 3, 115 + 5, and 115 + 7 indicate 1, 3, 5, and 7 days of shelf life at room temperature (20 °C) after cold storage at 0 °C for 115 days.

The main phenolic components of the two pear cultivars are arbutin, chlorogenic acid, caffeic acid, catechin, epicatechin, rutin, and isorhamnetin-3-glucoside. In comparison to ‘Yali’, ‘Wujiuxiang’ has lower arbutin and isorhamnetin-3-glucoside contents in the peel but has higher catechin and epicatechin levels (Figure 4). The caffeic acid content did not vary significantly between the two cultivars. ‘Wujiuxiang’ had higher isorhamnetin and chlorogenic acid contents after day 7 of the shelf life (Figure 4).

**Figure 5:**
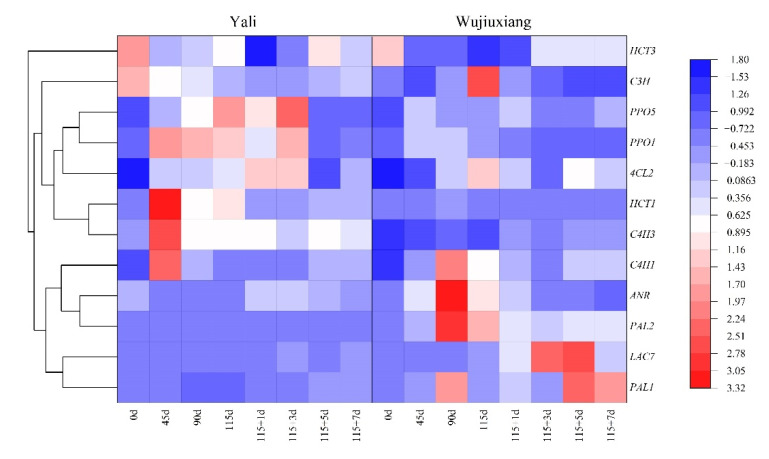
Expression profiles of genes involved in phenolic biosynthesis and metabolism in the peels of ‘Yali’ and ‘Wujiuxiang’ pears during cold storage and the shelf life. Note: 115 + 1, 115 + 3, 115 + 5, and 115 + 7 indicate 1, 3, 5, and 7 days of shelf life at room temperature (20 °C) after cold storage at 0 °C for 115 days. Red and high positive values indicate high gene expression, while blue and low negative values indicate low gene expression.

In terms of the expression patterns of the genes involved in phenolic synthesis, the *PAL1* and *PAL2* gene expression levels were significantly higher in ‘Wujiuxiang’ than in ‘Yali’, despite the fact that the expression patterns of *PAL1* and *PAL2* were different. When compared to ‘Yali’, the expression level of *ANR* in ‘Wujiuxiang’ was higher during cold storage and lower throughout the shelf life period. However, the expression levels of *HCT1* and *C4H3* in ‘Wujiuxiang’ were lower than in ‘Yali’, and the highest expression level in ‘Yali’ emerged in the early stage of cold storage (45 d). It should be noted that the expression of *C3H* in ‘Wujiuxiang’ was significantly higher than in ‘Yali’ only after 115 d. The *C4H1* expression was higher in ‘Wujiuxiang’ after 90 and 115 days of cold storage. The expression of *4CL2* was higher in ‘Wujiuxiang’ than in ‘Yali’ at days 115 and 115 + 5. In contrast to ‘Yali’, the expression of *HCT3* in ‘Wujiuxiang’ was significantly lower during cold storage and subsequently increased at 1 and 3 days of shelf life and finally decreased (Figure 5). The specific expression values are shown in Supplementary Table S2.

### 2.4. Changes in PPO and LAC Gene Expression

The *PPO1* and *PPO5* expression levels rose dramatically during cold storage (Figure 5). The *PPO1* expression level was lower in ‘Wujiuxiang’ throughout cold storage, as well as the shelf life period. The expression level of *PPO5* was decreased in ‘Wujiuxiang’ during 90–115 days of cold storage and the early shelf life (days 115 + 1115 + 3). Relatively speaking, the expression level of *LAC7* was lower than those of *PPO1* and *PPO5* and gradually increased after 90 days of cold storage, and then finally dropped after 7 days of shelf life in ‘Wujiuxiang’, which had significantly higher levels than ‘Yali’, with less variation in the expression (Figure 5).

### 2.5. Changes in Cell Membrane Permeability

**Figure 6:**
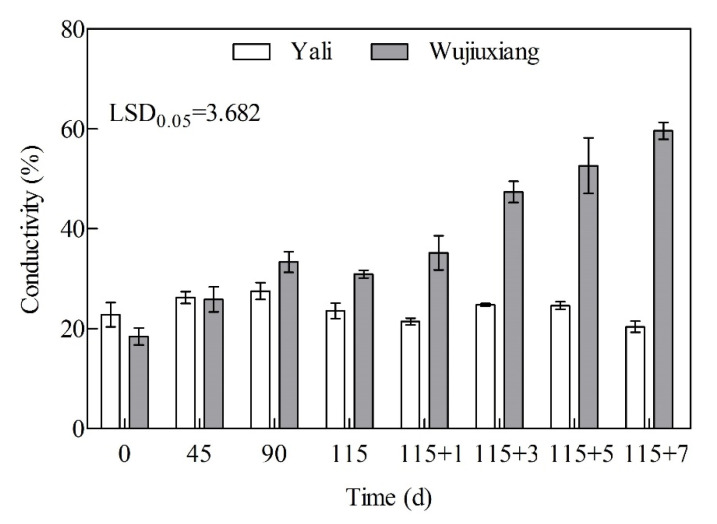
The relative conductivity of the peels of ‘Yali’ and ‘Wujiuxiang’ pear during cold storage and the shelf life. Vertical bar represents the standard error and LSD values indicate significant differences at the level of 0.05. Note: 115 + 1, 115 + 3, 115 + 5, and 115 + 7 indicate 1, 3, 5, and 7 days of shelf life at room temperature (20 °C) after cold storage at 0 °C for 115 days.

The relative conductivity is an indicator of cell membrane permeability. In ‘Wujiuxiang’, it increased gradually with the storage time and was significantly higher after 90 days of cold storage than in ‘Yali’, which exhibited less change (Figure 6).

### 2.6. PCA Analysis

**Figure 7:**
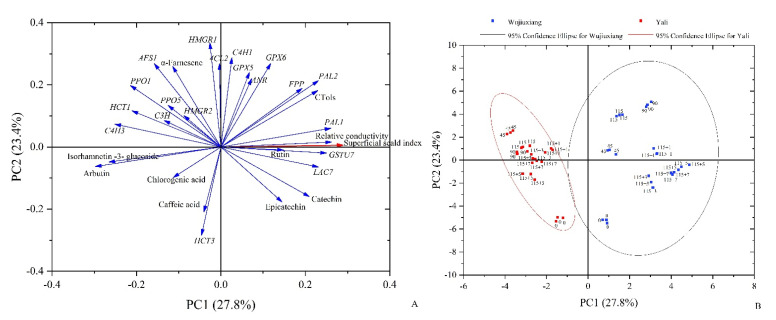
Loading plot (**A**) and score plot (**B**) of the principal component analysis (PCA) of the scald index, α-farnesene, CTols, and phenolic contents, as well as the related gene expression levels in ‘Yali’ and ‘Wujiuxiang’ pears. Note: arrows indicate the different variables. In the score plot, 0, 45, 90, 115, indicate the duration time of cold storage at 0 °C, 115 + 1, 115 + 3, 115 + 5, and 115 + 7, indicating 1, 3, 5, and 7 days of shelf life at room temperature (20 °C) after cold storage at 0 °C for 115 days.

The above-mentioned data were subjected to PCA analysis in order to acquire a better insight on the mechanism of superficial scald incidence in the two different pear cultivars. The total variability was explained by the first two principal components (PCs), with PC1 and PC2 accounting for 27.8% and 23.4% of the variation in the data, respectively. It was shown that the relative conductivity, rutin content, and *PAL1* and *GSTU7* expression levels were extremely positively correlated with the superficial scald index, and the CTols, catechin and epicatechin contents, as well as the *PAL2*, *FPP*, and *LAC7* expression levels, showed positive correlations as well, confirming their significant contributions to the development of scald (Figure 7A).

The score plot showed a clear separation of the samples according to the storage time (cold storage and shelf life) and cultivar (Figure 7B). For the ‘Yali’ pear, the samples (red squares) from different storage stages were mainly dispersed on PC2. However, the ‘Wujiuxiang’ samples (blue squares) from different storage times were scattered on both PC1 and PC2. Additionally, we observed the clearly different distribution of the ‘Yali’ and ‘Wujiuxiang’ pears on the score plot. This suggested a great distinction between the two pear cultivars in terms of superficial scald development.

## 3. Discussion

### 3.1. CTols Have a Great Influence on the Occurrence of Scald Compared to α-Farnesene

The accumulation of α-farnesene and its oxidation products, CTols, in pears is linked to the development of scald. Many studies have demonstrated that CTols play a more important role in the development of superficial scald than α-farnesene [4,19,28,29,30,31]. *GSTU7, GPX5*, and *GPX6* were key genes involved in the oxidation of α-farnesene to CTols [6]. The accumulation of CTols and higher expression levels of antioxidant genes, such as *GSTU7* and GPX6, were also linked to the incidence of superficial scald [6,16]. During cold storage, stress-associated, ROS-triggered *GSTU7* expression was activated, which may play a role in reducing peroxidation via glutathione (GSH) and producing scavengers of cytotoxic and genotoxic compounds [32]. *GPX6* was involved in senescence and abiotic stress [33,34]. Thus, this suggests that *GSTU7* and *GPX6* play crucial roles in the development of superficial scald in pear.

HMGR and AFS are key enzymes involved in the α-farnesene biosynthetic pathway. HMGR is the first enzyme involved in the synthesis of mevalonic acid (MVA). It has been identified that, among the *HMGR* genes, *HMGR2* is involved in α-farnesene synthesis and scald development in pear [6]. Furthermore, *AFS* is the final enzyme involved in the production of α-farnesene, and *AFS1* was found to be responsible for α-farnesene biosynthesis and scald [12]. In this study, the α-farnesene content was in accordance with the *AFS1* expression level in ‘Yali’, in contrast to ‘Wujiuxiang’, after 90 days (Figure 2). This is similar to the results of the ‘Blanquilla’ pear [19]. Therefore, the role of α-farnesene and *AFS* gene expression in the occurrence of superficial scald in pears should be investigated further. Meanwhile, in ‘Wujiuxiang’, the expressions of *HMGR1* and *HMGR2* were significantly upregulated during the development of superficial scald. However, the higher *FPP* expression and scald were found during the shelf life (Figure 3), accompanied by a decrease in the α-farnesene content (Figure 2), which can be linked with the PCA result (Figure 7) showing that the *FPP* expression and α-farnesene content were markedly different at different times, implying that the *FPP* expression was less closely related to the pattern of changes in the α-farnesene content, although it was related to the progression of superficial scald symptoms, which is consistent with the findings obtained for apples [18].

### 3.2. Phenols and Their Oxidation Reactions

PAL, C4H, 4CL, HCT, CHS, and ANR are key regulatory enzymes of the polyphenol biosynthetic pathway [35]. Previous studies have demonstrated that 1-methylcyclopropene (1-MCP) can downregulate the *PAL* and *C3H* gene expression, thus reducing the chlorogenic acid content and scald incidence [15]. The results showed that the expression levels of *PAL1*, *PAL2*, *C4H1*, *4CL2*, and *C3H* were higher, whereas the expression levels of *HCT1*, *HCT3* and *C4H3*, as well as the content of chlorogenic acid in the peel, was lower in the ‘Wujiuxiang’ pear (Figure 5). This indicates that the relationship between the expression of these genes and the chlorogenic acid content is not clear, similar to the results obtained for apples [17]. Additionally, ANR is the key enzyme that contributes to the biosynthesis of epicatechin [35]. In the present study, ‘Wujiuxiang’ showed a higher expression of *ANR* and more epicatechin than ‘Yali’ during cold storage (Figure 4 and Figure 5), and the epicatechin content decreased during storage (Figure 4C). These developments might result from the homeostasis of polyphenol synthesis and oxidation.

In apples and pears, chlorogenic acid, the PPO activity and its gene expression are involved in the development of superficial scald [11,27]. However, our study showed that the peel of the ‘Yali’ pear had more chlorogenic acid and a higher expression of *PPO1* and *PPO5*, but no scald occurred (Figure 1, Figure 4B and Figure 5). Similar phenomena were also found in the case of the ‘Conference’ pear [19]. Interestingly, it has been suggested that the PPO activity is not the rate-limiting factor in the internal browning (IB) of pear fruit [36], which suggests that chlorogenic acid and PPO are not the key limiting factors in scald development. On the other hand, higher contents of free phenolic compounds are more resistant to scald incidence in apples [37]. Therefore, the roles of chlorogenic acid and PPO in the onset and development of superficial scald are worthy of further study.

LAC is involved in superficial scald in apples and pears, as well as litchi browning [20,22,38,39]. Epicatechin is the reaction substrate of LAC [38,39], and LAC plays a key role in catalyzing the oxidation of epicatechin [38,39]. There was a higher epicatechin content and *LAC7* expression in ‘Wujiuxiang’ pear (Figure 4C and Figure 5), which is consistent with more advanced scald development. 

Due to the different cellular compartments of PPO and phenolic substances (such as chlorogenic acid), the integrity of the cell membrane structure is more crucial, and enzymatic browning may occur, followed by cell membrane injury [36,40]. In addition, the conductivity of ‘Wujiuxiang’ was much greater than that of ‘Yali’, and the cell membrane integrity/breakdown is strongly connected with the formation of superficial scald, with a rapidly increasing trend during storage (Figure 6).

## 4. Materials and Methods

### 4.1. Plant Materials

The ‘Wujiuxiang’ and ‘Yali’ pear fruits of uniform sizes (the average single fruit weight was 296.92 ± 15.17 g and 290.78 ± 7.30 g for ‘Wujiuxiang’ and ‘Yali’, respectively) were collected on August 29 and September 19, 2015, respectively, from 30-year-old trees and planted at 3 ×5 m on *Pyrus betulifolia* Bunge rootstock at an orchard (E114°58′20″ and N37°47′30″) located in Jinzhou City, Hebei Province, China. 

### 4.2. Experimental Design

The fruit was transported back to the laboratory and kept overnight at room temperature (25 ± 2 °C). Those with no pests and external damage were selected and then stored at a low temperature (0 °C) for 115 days. Then over their shelf life, they were observed after 1, 3, 5 and 7 day(s) at room temperature (20 °C), these periods being labelled as 115 + 1, 115 + 3, 115 + 5, and 115 + 7, respectively. After the superficial scald index was calculated for the intact fruit, the peel samples were immediately frozen in liquid nitrogen and kept at −80 °C until further use. 

### 4.3. Measurement of the Superficial Scald Index

The proportion of peel browning to the total peel area was divided into four grades, following Feng et al. [21]: grade 0, no browning; grade 1, 0% < browning area ≤ 25%; grade 2, 25% < browning area ≤ 50%; and grade 3, browning area > 50%. Each treatment included three replicates, and each replicate used 10 fruits.

The calculation formula is as follows:

Superficial scald Index = ∑ (scald level × number of fruit at the level)/(3 × total number of fruit)(1)

### 4.4. Determination of α-Farnesene and CTols Contents

The contents of α-farnesene and CTols were determined using Feng et al.’s method [21]. The peel disc (1 cm in diameter) was separated from the equatorial part of the fruit, and 20 discs per replication were placed in 25 mL test tubes. After that, 15 mL of hexane was added to the mixture, and the samples were kept in dark for 2 h. The extract was filtered using a clean Florisil SPE column and the absorbance was read at 232, 281, and 290 nm using a UV-Vis spectrophotometer (model: UV-2100, UNICO Instrument, Dayton, OH, USA). The α-farnesene and CTols were calculated using the molar extinction coefficients ε_232_ = 27,740 and ε_281–290_ = 25,000, respectively, and expressed as nmol cm^–2^. Each treatment included three replicates, and each replicate used 5 fruits.

### 4.5. Estimation of the Phenolic Component Contents

The contents of phenolic components were determined according to Schieber et al. [41]. We suspended two grams of the powdered frozen peel sample in 10 mL of 80% methanol and incubated the sample for 30 min in an ultrasonic oscillator. After centrifugation at 10,000 g for 10 min at 4 °C, the supernatant was collected and subjected to solid phase extraction using the C18 column, followed by washing with methanol and filtration through a 0.45 µm organic membrane. The 5 µL of filtrate was injected via high-performance liquid chromatography (HPLC) and read at 280 nm [42]. The HPLC (HITACHI L2000, Japan) device was equipped with an LaChrom C18 column (250 mm × 4.6 mm, 5 µm) in a mobile phase (5% acetic acid and acetonitrile) with a flow rate of 1 mL min^−1^ at 30 °C. The concentration of each phenolic compound was presented as μg g^−1^ FW.

### 4.6. Relative Conductivity Measurement

Peel discs with diameters of 1 cm were taken from the equatorial part of the fruit, and 25 discs were placed in 25 mL tubes in each replication [21]. After rinsing with 0.1 mol L^−1^ mannitol, the volume was set to 25 mL by mannitol and then shaken slightly at 25 °C for 2 h. The conductivity (C1) of the extract was measured using the DDS-307 conductivity meter (INESA Auto Electronics System Co., Ltd., Shanghai, China). Afterwards, the conductivity (C2) was measured again using extract cooled at 25 °C after being placed in the boiling water bath for 15 min. The relative conductivity was calculated as the percentage of C1 to C2. Each treatment included three replicates and each replicate used 5 fruits.

### 4.7. RNA Isolation and Real-Time PCR (RT-PCR) Analysis

One hundred milligrams of grounded peel sample were used for the RNA isolation using the RNA Prep Pure Plant Plus Kit (Polysaccharides & Polyphenolics-rich) (Tiangen Biotech Co., Ltd., Beijing, China). After the electrophoretic analysis, the first strand of cDNA was synthesized by reverse transcription using the PrimeScript RT reagent kit with a gDNA eraser (Perfect Real Time) (Takara Bio Inc., Dalian, China), and three replications were conducted for each sample. RT-PCR analysis was performed on an ABI 7500 Real-Time System (Applied Biosystems, Foster City, CA, USA) using the SYBR Premix Ex Taq^TM^ kit (Takara Bio Inc., Dalian, China). The expression of related genes was defined as 1.0 using pear *PbACTIN2* as the internal reference gene [43], and the relative quantitative expression was obtained using the method of 2^-ΔΔCT^. The sequences of the RT-PCR primers [6,18,20,27,43,44,45] are shown in Supplementary Table S1.

### 4.8. Statistical Analysis

The data were analyzed using SPSS 18 software (SPSS Inc., Chicago, IL, USA), and ANOVA was used to generate least significant differences (LSD) (*p* < 0.05). Figures were prepared using GraphPad Prism 4 (GraphPad Software Inc., La Jolla, San Diego, CA, USA). Origin 9.0 (OriginLab Co., Northampton, MA, USA) was used to create heatmaps and perform the PCA analysis.

## 5. Conclusions

‘Wujiuxiang’ (sensitive to scald) and ‘Yali’ (less sensitive to scald) pears showed varying sensitivity to superficial scald during cold storage, as well as the shelf life. In contrast to the ‘Yali’ pear, the expression of genes involved in α-farnesene and CTols metabolism (*HMGR1*, *HMGR2*, *GSTU7*, *GPX5*, and *GPX6*) and in phenolic biosynthesis (*PAL1*, *PAL2*, *C4H1*, *4CL2*, *C3H*, and *ANR*) was significantly upregulated upon the onset of superficial scald. Furthermore, the relative conductivity and the contents of catechin and epicatechin were higher, and the expression levels of related genes (*LAC7* and *ANR*) significantly increased with the development of superficial scald, whereas the lower contents of chlorogenic acid, arbutin, and isorhamnetin-3-3-glucoside, as well as the lower expression levels of *C4H3*, *HCT3*, *PPO1*, and *PPO5*, were found in the peel of the ‘Wujiuxiang’ pear. The accumulation of CTols, cell membrane disintegration, and the oxidation process of phenols in pear fruit were all linked to the onset and development of superficial scald.

## Data Availability

Data are contained within the article or the Supplementary Materials.

## Supplementary Materials

The supporting information can be downloaded at: https://www.mdpi.com/article/10.3390/ijms232012088/s1. References [6,18,20,27,43,44,45] are cited in the supplementary materials.
