# Supplementary material for: Changes in α-Farnesene and Phenolic Metabolism and the Expression of Associated Genes during the Development of Superficial Scald in Two Distinct Pear Cultivars
Source: Int J Mol Sci. 2022 Oct 11;23(20):12088. doi: 10.3390/ijms232012088 (PMC9603056; doi:10.3390/ijms232012088)
Supplement: Supplementary file 1 [file ijms-23-12088-s001.zip › Supplementary Table S1.pdf]

Table S1. Primer sequence for RT-PCR quantitative analysis

Gene	Genbank ID	Sequence (5'-3')	Source of primers
ACTIN2	GU830959	GGACATTCAACCCCTCGTCT<br>ATCCTTCTGACCCATACCAACC	[43]
AFS1	DQ309034	AATGGTTGGAACCAAGTATTACC<br>GAAACTGATGATGAATCGCATCC	[6]
HMGR1	XM_009360626	GTTCTCACTGCATTACCATG<br>TCAGACAAGCGGATTGAGATG	
HMGR2	KF861867	CAGTTGGAGGAGGAACCCAG<br>AGTTTGAACCGGGTGAGTC	
GSTU7	XM_009372621	ATGGTTCCGGTGTTTGTCCA<br>GAGGGTCATTTGGCAGCAGA	
GPX5	XM_009349430	GGGACAAGTCAGGATGCTGAAC<br>ACAGGTTCTGTATCCGGCCCA	
GPX6	XM_009341641	AGACCCTTTTGCCACGGTCG<br>CGGACTGGCCAGCCATTGTA	
FPP	XM_009349237	GAAATCTGAGCTTCTCAATGATCC<br>CCTCGATTCAGCTTTCCTCC	[18]
PAL1	GU906268	GACGCACAAGTTGAAGCACC<br>TTTGACATAAGAGCTGCCATCC	[27]
PAL2	GU906269	ACCAAAAGAAGGGCTTGCCT<br>AAAGCGCTTGTGTGTTAGCG	
C4H1	XM_009376113	AACTTCGAGCTTCTGCCTCC<br>CCCCAAGCATCAATCTACGC	
C4H3	XM_009356605	AACTTGGAGCTTCTGCCTCC<br>AAAGAAATCATTAATCTACGCCCTC	
4CL2	XM_009353551	AATTCAAAGGCTTCCAAGTGC<br>CACTAGACCGAACCACAAATGC	[44]
C3H	XM_009355500	TTACAAGCCGTGCCTACTCC<br>CACAAAAACAAGAAACACTACGG	[27]
HCT1	JQ280303	CCCCCTCCAGTCTGACCA<br>CCAATGAAAACACAAACACGTC	
HCT3	NM_001302311	ACGATATGCGTGAGAAGTTTATCC<br>AGCACTAGGAAAGAAACACGACC	[44]
ANR	XM_009370102	GTAGCCCTTCTGGAGCAGC<br>ACAACCCATCTCAAAGAAGACG	[45]
PPO1	HQ729709	TCCCTACTCACAAAGCCCAAG<br>GACCTCCAAGACCAAGAAGCA	[27]
PPO5	GU906266	ACCAAAACAAAAACCATTCCAC<br>CAGCCACTCCACCATACAGG	
LAC7	XM_009348720	TCATCAACCCCCATTATGC<br>GAACATGTGCTCGTCCACC	[20]
