# Supplementary material for: Changes in α-Farnesene and Phenolic Metabolism and the Expression of Associated Genes during the Development of Superficial Scald in Two Distinct Pear Cultivars
Source: Int J Mol Sci. 2022 Oct 11;23(20):12088. doi: 10.3390/ijms232012088 (PMC9603056; doi:10.3390/ijms232012088)
Supplement: Supplementary file 1 [file ijms-23-12088-s001.zip › Supplementary Table S2.pdf]

Table S2. Relative expression amounts ($2^{-\Delta\Delta CT}$) of α-farnesene and CTols biosynthesis-related genes, phenolics biosynthesis and metabolism genes in peels of 'Yali' and 'Wujiuxiang' pears during cold storage and shelf-life.

Note: The 115 + 1, 115 + 3, 115 + 5, and 115 + 7 indicated as 1, 3, 5 and 7 days of shelf life at room temperature (20 °C) after cold storage at 0 °C for 115 days.

Values are presented as mean ± standard deviation, different small letters shows significant differences at the 0.05 level.

Gene	Time (d)	0 d	45 d	90 d	115 d	115+1 d	115+3 d	115+5 d	115+7 d
GSTU7	Yali	1.00±0.07 i	1.57±0.11 hi	3.37±0.26 ghi	5.51±0.39 fg	27.61±1.98 d	25.99±1.19 d	4.88±0.56 fgh	7.10±0.69 f
	Wujiuxiang	1.74±0.58 hi	5.723±2.379 fg	5.37±1.23 fg	20.21±1.78 e	114.03±7.64 b	53.17±5.50 c	110.13±7.71 b	127.14±7.94 a
FPP	Yali	1.00±0.13 k	5.98±0.29 de	5.77±0.09 ef	4.88±0.09 g	7.33±0.22 b	3.03±0.11 j	4.07±0.01 h	3.58±0.07 i
	Wujiuxiang	3.80±0.13 hi	6.25±0.16 d	6.25±0.17 d	6.14±0.22 d	8.79±0.21 a	5.53±0.14 f	8.56±0.23 a	6.83±0.10 c
HMGR2	Yali	1.00±0.08 c	0.93±0.03 c	0.55±0.06 fg	0.49±0.02 g	0.50±0.02 g	1.12±0.11 b	1.09±0.05 b	1.38±0.06 a
	Wujiuxiang	0.37±0.02 h	0.57±0.02 efg	0.93±0.09 c	1.30±0.05 a	0.62±0.03 def	0.52±0.01 g	0.68±0.02 d	0.66±0.01 de
GPX6	Yali	1.00±0.02 l	8.27±0.06 c	4.53±0.06 h	3.88±0.09 i	3.27±0.08 j	3.37±0.08 j	2.92±0.08 k	3.41±0.01 j
	Wujiuxiang	1.20±0.01 l	12.20±0.63 a	10.32±0.04 b	7.27±0.05 d	6.66±0.17 e	3.78±0.07 i	5.49±0.14 g	5.95±0.10 f
GPX5	Yali	1.00±0.02 de	1.07±0.04 d	0.95±0.04 def	0.85±0.02 f	0.64±0.02 h	0.71±0.03 gh	0.71±0.02 gh	0.65±0.08 h
	Wujiuxiang	0.82±0.03 fg	1.83±0.00 c	3.61±0.21 a	2.76±0.17 b	0.89±0.02 ef	0.50±0.01 i	0.48±0.02 i	0.38±0.00 i
HMGR1	Yali	1.00±0.02 l	7.04±0.28 b	5.14±0.02 d	5.02±0.05 d	4.34±0.18 f	2.39±0.13 ij	2.51±0.11 hi	2.52±0.01 hi
	Wujiuxiang	1.65±0.04 k	4.78±0.06 e	8.65±0.04 a	6.79±0.04 c	3.47±0.04 g	1.82±0.02 k	2.70±0.21 h	2.24±0.06 j
AFS1	Yali	1.00±0.07 k	30.71±2.00 a	21.07±1.11 cd	20.51±0.26 cde	8.96±0.23 hi	13.55±1.46 g	19.98±0.41 de	21.52±0.58 c
	Wujiuxiang	0.34±0.02 k	19.35±0.53 e	23.16±0.09 b	14.81±0.43 f	7.77±0.16 i	6.28±0.09 j	9.59±0.20 h	9.21±0.28 h

HCT3	Yali	1.00±0.04 a	0.58±0.02 f	0.63±0.02 ef	0.72±0.01 cd	0.20±0.00 i	0.47±0.05 g	0.78±0.03 c	0.61±0.03 ef
	Wujiuxiang	1.19±0.62 b	0.42±0.00 gh	0.42±0.02 gh	0.28±0.03 i	0.36±0.02 h	0.73±0.19 de	0.68±0.10 de	0.68±0.01 de
C3H	Yali	1.00±0.04 b	0.77±0.01 c	0.62±0.02 d	0.52±0.02 e	0.39±0.01 f	0.44±0.02 f	0.53±0.02 e	0.56±0.02 e
	Wujiuxiang	0.31±0.01 g	0.17±0.02 i	0.44±0.01 f	1.37±0.10 a	0.43±0.03 f	0.23±0.01 h	0.13±0.01 i	0.13±0.01 i
PPO5	Yali	1.00±0.03 l	126.61±1.40 g	221.14±3.07 d	373.73±8.10 b	252.52±8.62 c	451.79±27.88 a	6.18±0.05 l	5.77±0.32 l
	Wujiuxiang	1.47±0.11 l	162.77±3.39 e	80.15±2.01 j	95.42±7.79 j	145.52±3.96 f	49.75±13.20 k	57.53±8.23 k	110.77±16.42 h
PPO1	Yali	1.00±0.04 j	1604.70±9.64 a	1462.87±18.84 b	1424.57±21.14 c	856.90±8.58 d	1471.28±46.59 b	104.61±0.33 i	140.42±7.03 h
	Wujiuxiang	0.91±0.03 j	761.15±5.14 e	727.84±10.59 f	407.01±46.10 g	164.86±6.16 h	24.70±0.93 j	23.48±1.80 j	24.62±0.78 j
4CL2	Yali	1.00±0.01 h	9.52±0.05 cd	9.34±0.20 cd	10.60±0.21 b	13.94±0.31 a	14.13±0.98 a	3.37±0.10 g	8.17±0.04 e
	Wujiuxiang	1.44±0.05 h	3.91±0.05 g	9.62±0.07 cd	14.36±1.01 a	9.84±0.27 c	5.35±1.20 f	11.23±1.60 b	8.99±0.15 d
HCT1	Yali	1.00±0.21 g	25.12±2.83 a	8.67±1.14 b	9.77±1.78 b	3.20±0.45 cde	2.92±0.63 def	4.70±0.20 c	4.57±0.38 cd
	Wujiuxiang	0.73±0.03 g	1.19±0.16 fg	2.17±0.20 efg	0.95±0.04 g	1.72±0.21 efg	0.95±0.40 g	1.35±0.41 fg	1.41±0.24 efg
C4H3	Yali	1.00±0.08 d	2.97±0.25 a	1.67±0.19 b	1.68±0.10 b	1.65±0.14 b	1.43±0.13 c	1.78±0.08 b	1.59±0.09 bc
	Wujiuxiang	0.34±0.02 g	0.36±0.01 g	0.66±0.08 f	0.52±0.07 fg	0.89±0.06 de	0.72±0.09 ef	0.99±0.22 d	0.92±0.09 de
C4H1	Yali	1.00±0.01 h	3.23±0.07 a	1.63±0.03 cde	1.39±0.00 fg	1.31±0.05 g	1.26±0.03 g	1.59±0.01 def	1.59±0.08 def
	Wujiuxiang	0.72±0.01 i	1.53±0.06 ef	3.03±0.17 a	2.16±0.22 b	1.70±0.07 cde	1.26±0.28 g	1.81±0.27 cd	1.84±0.04 c
ANR	Yali	1.00±0.04 e	0.44±0.01 gh	0.52±0.03 fgh	0.53±0.01 fgh	1.31±0.06 d	1.19±0.04 d	0.95±0.04 e	0.66±0.03 f
	Wujiuxiang	0.59±0.02 fg	1.48±0.06 c	4.29±0.23 a	2.09±0.20 b	1.22±0.02 d	0.38±0.08 h	0.43±0.04 h	0.17±0.00 i
PAL2	Yali	1.00±0.08 g	0.14±0.03 g	0.08±0.01 g	0.09±0.01 g	0.42±0.01 g	0.04±0.00 g	0.09±0.00 g	0.04±0.01 g
	Wujiuxiang	1.57±0.08 g	18.36±0.21 f	121.32±2.65 a	71.10±8.48 b	36.60±0.93 d	25.06±1.20 e	41.76±5.56 c	34.67±1.33 d

LAC7	Yali	1.00±0.10 ef	0.23±0.03 ef	0.11±0.00 f	0.17±0.03 f	1.12±0.23 ef	1.44±0.04 ef	0.57±0.05 ef	1.67±0.02 ef
	Wujiuxiang	0.74±0.11 ef	0.57±0.06 ef	1.00±0.16 ef	2.46±0.27 e	13.65±0.48 c	34.66±0.12 b	36.01±4.64 a	10.28±0.75 d
PAL1	Yali	1.00±0.02 ef	0.65±0.03 fg	0.25±0.01 g	0.22±0.01 g	0.40±0.01 g	0.43±0.01 g	1.31±0.02 de	1.75±0.02 d
	Wujiuxiang	0.48±0.02 g	1.12±0.02 ef	6.92±0.16 b	1.37±0.10 de	2.92±0.13 c	1.69±0.38 d	8.74±0.95 a	7.16±0.41 b
